# Structural insights into bacterial flagellar hooks similarities and specificities

**DOI:** 10.1038/srep35552

**Published:** 2016-10-19

**Authors:** Young-Ho Yoon, Clive S. Barker, Paula V. Bulieris, Hideyuki Matsunami, Fadel A. Samatey

**Affiliations:** 1 Trans-Membrane Trafficking Unit, Okinawa Institute of Science and Technology Graduate University, 1919-1, Tancha, Onna, Kunigami, Okinawa 904-0495, Japan

## Abstract

Across bacteria, the protein that makes the flagellar hook, FlgE, has a high variability in amino acid residue composition and sequence length. We hereby present the structure of two fragments of FlgE protein from *Campylobacter jejuni* and from *Caulobacter crescentus*, which were obtained by X-ray crystallography, and a high-resolution model of the hook from *Caulobacter*. By comparing these new structures of FlgE proteins, we show that bacterial hook can be divided in two distinct parts. The first part comprises domains that are found in all FlgE proteins and that will make the basic structure of the hook that is common to all flagellated bacteria. The second part, hyper-variable both in size and structure, will be bacteria dependent. To have a better understanding of the *C. jejuni* hook, we show that a special strain of *Salmonella enterica*, which was designed to encode a gene of *flgE* that has the extra domains found in FlgE from *C. jejuni*, is fully motile. It seems that no matter the size of the hook protein, the hook will always have a structure made of 11 protofilaments.

Gram-positive and gram-negative bacteria use flagella for propulsion^1^. Flagella can be divided into three major structural parts: the filament, the hook, and the basal body containing the motor. The hook, a highly flexible universal joint that connects the filament to the motor, consists of about 120 copies of a single protein, FlgE^2^,^3^. Extensive studies on bacterial flagella show that the filament and the hook make helical assemblies best described as tubular structures comprising 11 protofilaments^4^,^5^. Quaternary organization of the hook and the filament has long been assumed to be similar for all bacterial flagella^6^,^7^. However, cryo-electron microscopy experiments with *Campylobacter jejuni* (*C. jejuni*) have showed that its filament has only seven protofilaments^8^. The degree of divergence of FlgE sequences in the bacterial kingdom brings various questions about the quaternary structure of the hook in general and that of *C. jejuni* in particular. We examined the structures of large inserts in the FlgE sequence by solving the structures of FlgE from *Caulobacter crescentus* (*C. crescentus*) and *C. jejuni*.

## Results

### Structure of the FlgE protein from *Caulobacter* and *Campylobacter*

*C. crescentus* strain CB15 (ATCC 19089) has one gene, CC_0902, encoding a 591 amino acid hook protein FlgE (61 kDa). We crystallized a 32 kDa fragment of FlgE. This fragment, called FlgEcc32, lacks the N-terminal 166 residues and the C-terminal 122 residues.

*C. jejuni* strain NCTC 11168 possesses two hook genes, *Cj0043* and *Cj1729c*, encoding two distinct hook proteins, FlgE (58 kDa) and FlgE2 (92 kDa), respectively. However, it was found by purifying and characterizing the composition of the *C. jejuni* flagellar hook that it is composed of only FlgE2^9^. *C. jejuni* FlgE2, hereafter termed FlgEcj, with 865 amino acid residues, is one of the longest bacterial flagellar hook proteins. We crystallized a 79 kDa fragment of FlgEcj. This fragment, called FlgEcj79, lacked the N-terminal 90 residues and the C-terminal 37 residues.

We solved the structures of both FlgEcc32 and FlgEcj79 to resolutions of 1.84 Å and 2.45 Å (Table 1), respectively, using X-ray crystallography. Both FlgEcc32 and FlgEcj79 contain completely different extra domains that are not present in FlgE from *Salmonella enterica*.

**Figure 1:**
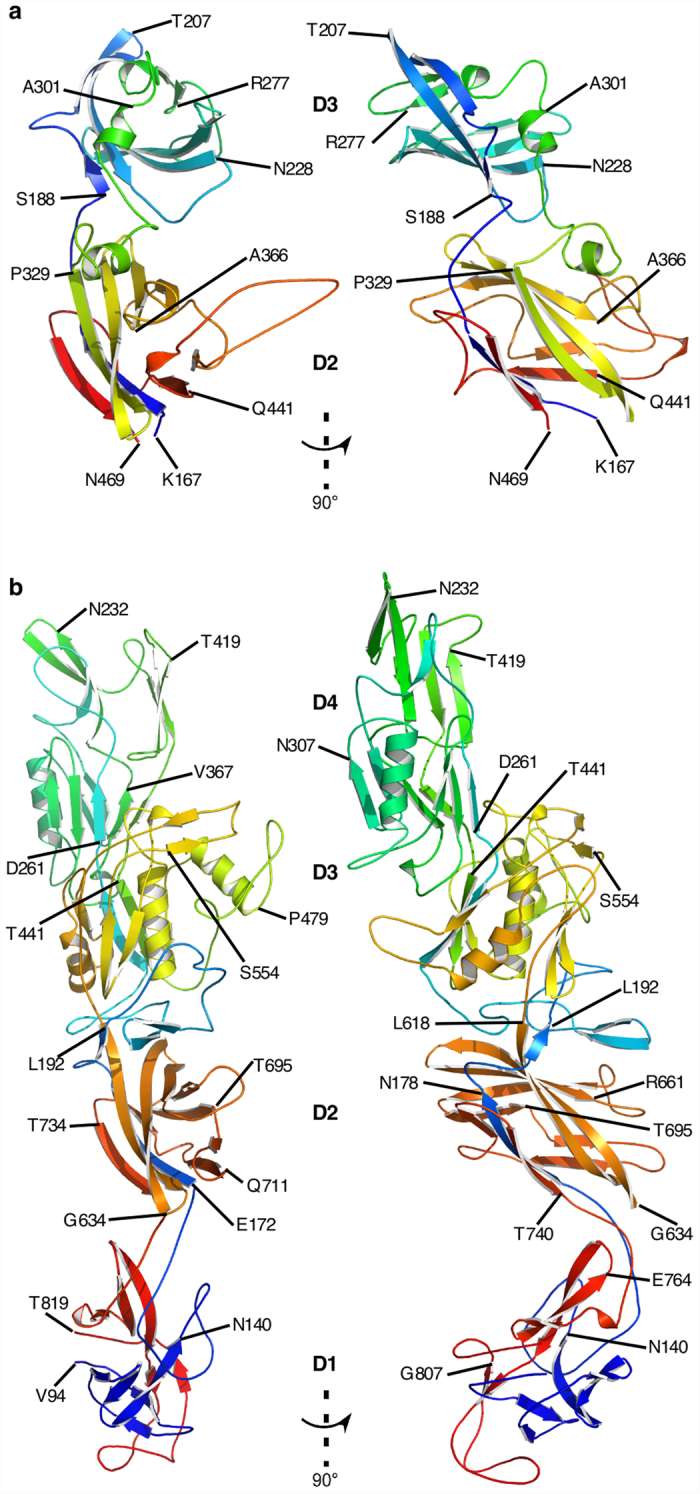
Structure of FlgE proteins from *C. crescentus* and *C. jejuni.* (**a**) Views of the Cα backbone of FlgEcc32 with its 2 domains, D2 and D3. (**b**) Two different views of the Cα backbone of FlgEcj79 with its 4 domains, D1, D2, D3, and D4. The chains are colour-coded from the N- to the C-termini in rainbow colour sequence from blue to red.

The Cα backbone trace of FlgEcc32 of *C. crescentus* (Fig. 1a) shows two domains, D2 and D3, based on the distribution of hydrophobic amino acids (Supplementary Fig. S1a). Domain D2 consists of β-strands organized like a rugby ball, that is completed with some long loops. This domain is made of two parts that consist of segments Lys167-Val183 and Pro329-Asn469. Domain D2 has a loop that protrudes away from it, while Domain D3 consists of a single segment, Ala184-Lys328. It contains a half-barrel made of β-strands that is completed by an array of short α-helices and loops.

In the case of the structure of the hook protein of *C. jejuni*, the Cα backbone trace of FlgEcj79 shows four domains, D1, D2, D3, and D4, based on the distribution of hydrophobic amino acids (Supplementary Fig. S1b). Domain D4 is made from one continuous polypeptide segment. D1, D2, and D3 are each made from two discontinuous segments. The chain starts with Val94 in domain D1. Almost half of domain D1 is formed before the chain passes into domain D2, forming a six-residue β-strand (Glu172-Asn178). Then the chain continues into D3 in a long segment, Leu192-Ala260, that forms a “two β-turns - β-hairpins - α-helix” motif that ends with a β-strand, all connected by long loops. The chain then forms domain D4, after which it returns to complete domains D3, D2, and finally, the second part of D1, where it ends at the C-terminus with Thr819 (Fig. 1b). Domain D1 consists of the N-terminus Val94-Ala168 and the C-terminus Thr744-Thr819. It contains an intricate set of β-strands joined by loops having no specific secondary structure. Domain D2 consists entirely of β-strands organized like a rugby ball. It consists of Lys169-Leu192 and Leu618-Tyr743. Domain D3 consists of Tyr193-Ala260 and Thr441-Leu618. The core of domain D3, which consists of segments Ile440-Ser448, Asn496-Val518 and Gln533-Gln617, has a set of eight β-strands separated into two groups by two α-helices. Altogether, these β-strands and α-helices form an α-helix/β-strand barrel (αβ-barrel) that makes an angle of about 80° with domain D2. The remaining part of domain D3 consists of β-strands and α-helices loosely connected by long loops with no specific secondary structure. Domain D4 can be divided into two sub-domains (Supplementary Fig. S1b); it is made of a continuous segment, Asp261-Ile440. Sub-domain D4a, next to D3, contains a set of anti-parallel β-strands completed with one α-helix. Sub-domain D4b, the farthest from D3, has only anti-parallel β-strands connected by long loops.

### Comparison with the structure of FlgE from *Salmonella*

A comparison of known FlgE protein structures was made to understand the extent of differences and similarities between these bacterial species. The structure of FlgE from *S. enterica* (FlgEst) has been previously solved by X-ray crystallography^10^ and by electron cryo-microscopy^11^. Amino acid sequence alignments for FlgEst show an identity of 34% with *C. jejuni* FlgE, 27% with *C. crescentus* FlgE, and 26% between *C. crescentus* and *C. jejuni* FlgE (Supplementary Fig. S2). FlgEst (42 kDa) consists of 3 domains labeled D0, D1, and D2. D0 is made of the coiled-coil domain found in all flagellar axial proteins. It is essential for polymerization into the hook structure. Domains D1 and D2 make the inner and outer layer of the hook, respectively, and are involved, through their interactions, in the function of the hook as a universal joint. For the crystallographic study of FlgEcj79 and FlgEcc32, domain D0 of *C. jejuni* and domains D0 and D1 of *C. crescentus* were removed. The structures of FlgEcj79 domains D1 and D2 are very similar to those of FlgEst^11^. The overall structural alignment of domains D1-D2 from FlgEcj79 and FlgEst (PDB code 1WLG) gives a root mean square deviation (RMSD) of 2.7 Å. When we compared domains D1 and D2 separately, each aligned almost perfectly with the respective domains of FlgEst (Supplementary Fig. S3a and b). The two D1 domains superimpose with an RMSD of 1.4 Å. The major difference between the two D1 domains is the existence a short α-helix in *C. jejuni* FlgEcj that does not exist in *S. enterica.* The presence of this short α-helix, K749 - I753, is due to an insertion at this position (Supplementary Fig. S2). Domains D2 of FlgEcj79 and FlgEst superimpose with an RMSD of 1.6 Å. Similarly, domains D2 of FlgEcc32 and FlgEst superimpose with an RMSD of 1.7 Å (Supplementary Fig. S3c), and domains D2 of FlgEcc32 and FlgEcj79 superimpose with an RMSD of 0.9 Å (Supplementary Fig. S3d). D2 regions known to be involved in interactions between the hook protofilaments are preserved both in *C. crescentus* and in *C. jejuni* FlgE^5^,^10^.

Sequence alignment of *C. jejuni* FlgEcj and *S. enterica* FlgEst shows long stretches of amino acid insertions that correspond exactly to domains D3 and D4 of *C. jejuni* FlgEcj, which do not exist in *S. enterica* or *C. crescentus* (Supplementary Fig. S2). Domains D3 of FlgEcj and FlgEcc are located at the same position, relative to domain D2. However, their respective sequence is not conserved and their 3D folding is completely different. Structural similarity searches performed with DALI^12^ on domains D3 and D4 of FlgEcj79, and on domain D3 of FlgEcc32 indicate that these domains have a unique combination of structural motifs that are not found in the Protein Data Bank (PDB).

### Partial model of the hook from *Caulobacter*

**Figure 2:**
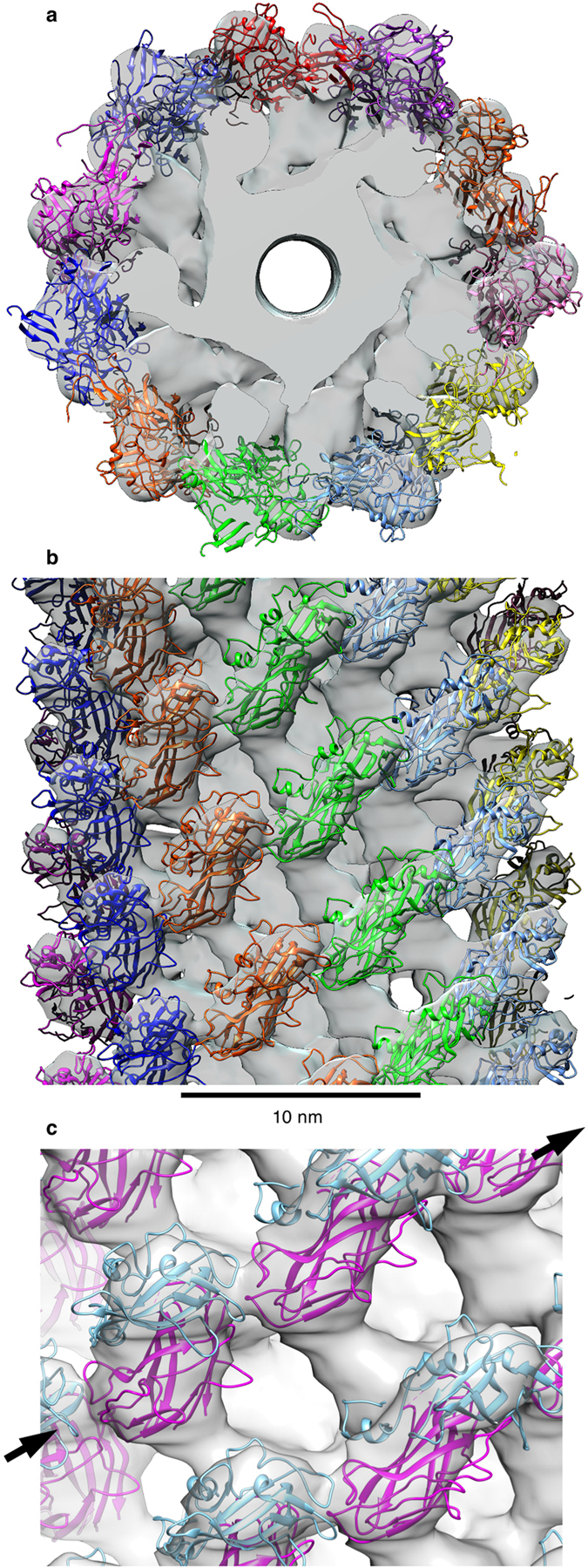
Manual docking of domains D2 and D3 of the hook protein, FlgE, of *C. crescentus* into the density map of the hook obtain by electron microscopy. (**a**) Top view from the distal end. Each protofilament has a different colour. The central channel is visible in the middle. (**b**) Side view of the hook with domains D2 and D3. Each colour represents a different protofilament. Domains D0 and D1, which are missing in the crystal structure, should fill the unoccupied density in the map of the hook. (**c**) D3-D2 domain interaction in the model of the hook protofilament. Interaction in the 6-start direction indicated by the black arrow between D3 (cyan) and D2 (magenta) domains.

The structure of the hook of *C. crescentus* had been solved at 20 Å resolution by electron microscopy^13^. Early comparison between density maps of the hook of *S. enterica* and of *C. crescentus*, obtained by electron microscopy, showed that the density corresponding to domain D3 of FlgE from *C. crescentus* was located in the outer part of the hook and with a high probability of contributing to a 6-start helical interaction in the hook^3^,^5^,^14^. To make a model of the hook, we manually docked the atomic model of FlgEcc32 into the density map of the hook of *C. crescentus* (Fig. 2). The helical parameters of the hook from *C. crescentus* were applied to the docked molecule to create a model of the hook with domains D2 and D3. The relative positions of domains D2 and D3 of FlgEcc32 need to be slightly rearranged to obtain a relatively good docking given the low-resolution of the map (Supplementary Fig. S4). The structure of the hook with domains D2 and D3 shows the eleven protofilaments that make the structure. This hook has a diameter of 245 Å, one-third larger than the 180 Å diameter of the hook of *S. enterica.* The main interactions found in the hook of *C. crescentus* are in the 6-start direction between D2-D2 domains as found in the hook of *S. enterica*^10^. However, we also have an interaction in the 6-start direction between D3-D2 of the neighbouring protofilaments (Fig. 2c).

### A *Salmonella* strain encoding an *S. enterica*-*C. jejuni* chimeric FlgE produces normal flagella

FlgEcj of *C. jejuni* is much larger than *C. crescentus* FlgEcc, and contains an insertion corresponding to domains D3 and D4 of approximately 386 amino acids, that is not found in *S. enterica* FlgEst. Based on sequence and structural alignments of FlgEst and *C. jejuni* FlgEcj79 we predicted that it should be possible to insert domains D3 and D4 from *C. jejuni* FlgEcj into the D2 domain of *S. enterica* FlgEst and for the hybrid FlgE protein to build a hook with the same 11-protofilament quaternary organization as the wild-type *Salmonella* flagellar hook. To examine this possibility a strain of *Salmonella* was made, TMT233a, bearing a hybrid *flgE* gene with codons for domains D3 and D4 of *C. jejuni* FlgEcj inserted into the *flgE* gene. The hybrid hook protein was predicted to be 84 kDa and consists of residues Gly233 to Leu618 of *C. jejuni* strain NCTC 11168 FlgE inserted between residues Thr167 and Pro168 of *S. enterica* FlgEst (Supplementary Fig. S5).

**Figure 3:**
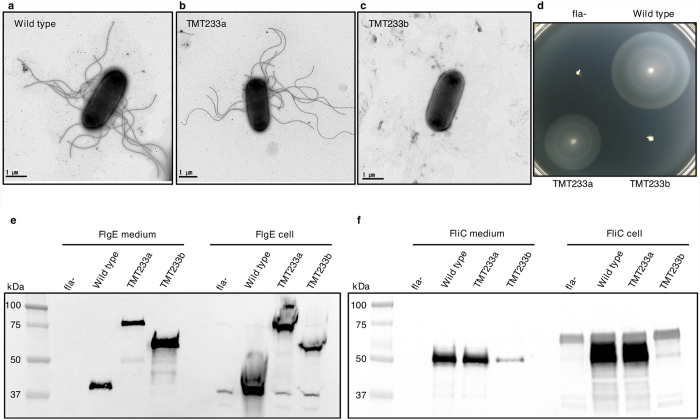
Flagellar biosynthesis for *Salmonella* strains producing a wild-type or hybrid FlgE hook protein. Negatively-stained TEM images of whole cells of *Salmonella* strains: Wild type (**a**), TMT233a (**b**) and TMT233b (**c**), TMT233a encodes a hybrid hook protein, FlgE_hyb1_, with codons for the D3 and D4 domains of *C. jejuni* FlgE inserted into the *flgE* gene. TMT233b encodes a hybrid hook protein, FlgE_hyb2_, with codons for the D3 domain of *C. jejuni* FlgE inserted into the *flgE* gene. Six-hour swimming assay on 0.3% (w/v) agar-tryptone plates with wild-type *S. enterica*, TMT233a and TMT233b (**d**). Western blots showing levels of FlgE (**e**) and of FliC (**f**) exported into the culture medium and expressed in cells of the wild-type *S. enterica*, TMT233a and TMT233b. The MW of wild-type FlgE is 42 kDa, FlgE_hyb1_ is 84 kDa, FlgE_hyb2_ is 65 kDa and FliC is 52 kDa. “fla-” indicates the non-flagellated negative control, which was a *flgE* mutant strain bearing a *tetRA* insertion (TMTflgEtetRA).

Transmission electron microscopy showed that negatively-stained cells of the strain encoding the FlgE chimera had about the same number of flagellar filaments as wild-type *Salmonella* (Fig. 3a,b) and it was almost as motile as wild type in soft-tryptone 0.35% (w/v) agar plates (Fig. 3d) and in liquid medium (Supplementary Movie S1). This indicates that normal flagella were made with the hybrid hook protein. We attribute the small decrease in swimming ability of the strain encoding the chimeric FlgE to the fact that the hook made of the chimeric FlgE with domains D3 and D4, lacks some of the flexibility of the wild-type *S. enterica* hook. Domains D3 and D4 will most likely increase the number of interactions between FlgE proteins in the hook and these new interactions may make the hook slightly stiffer. *Salmonella* cells produce peritrichous flagella over the cell surface, while *C. jejuni* cells produce monotrichous or amphitrichous flagella at one or both cell poles, respectively. For bacteria with peritrichous flagella, the hook needs to be very flexible to allow the flagella to form a helical bundle behind the cell during straight swimming phases, and also to allow a quick collapse of this bundle during tumbling of the cell before changing swimming direction^15^. This would be difficult with a stiffer hook. For the same reasons, when compared to wild-type *S. enterica*, our strain of *Salmonella*, TMT233a, bearing a hybrid *flgE* gene with codons for domains D3 and D4 of *C. jejuni* FlgEcj would not necessary swim better in viscous media. Free-swimming speeds of wild-type *S. enterica* and mutant strain TMT233a were measured in motility buffer containing increasing concentration of ficoll to increase solution viscosity, using a microscope with dark-field optics and video equipment (Supplementary Fig. S6). Without ficoll, strain TMT233a swam about two-thirds as quickly as wild type (13 versus 21 μm/sec). Swimming speeds of both strains decreased with increasing concentrations of ficoll.

**Figure 4:**
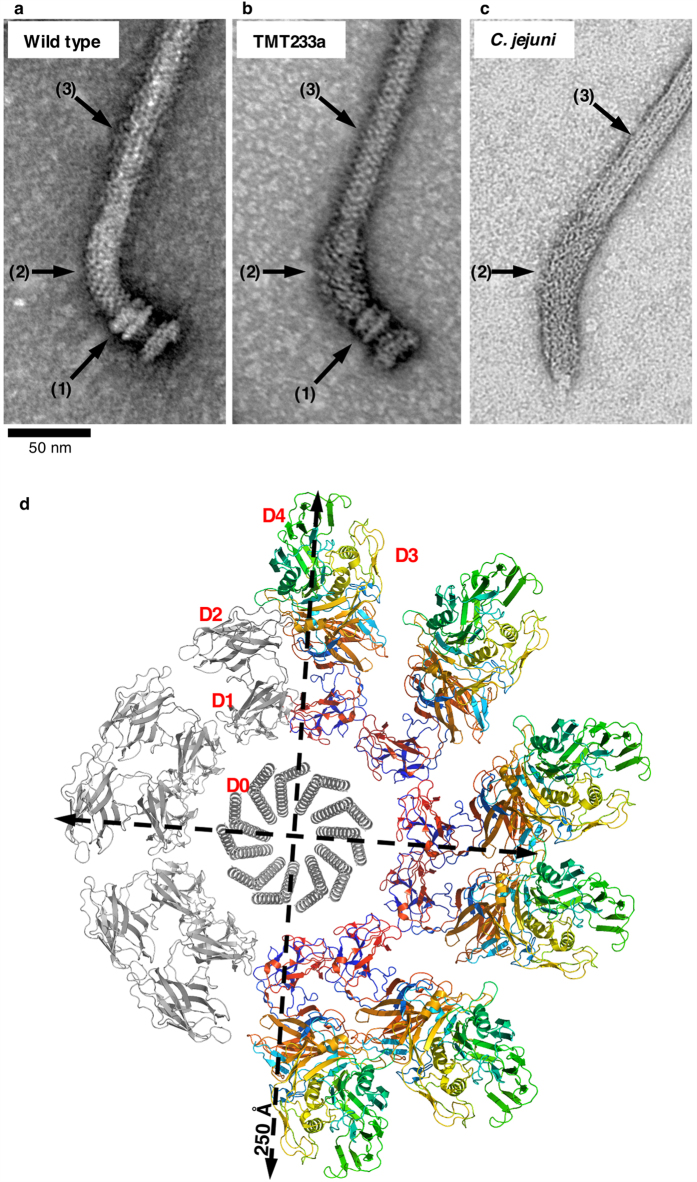
Transmission electron microscopy of purified flagella. Negatively-stained TEM images of a flagellum from *S. enterica* strain SJW1103 (**a**), which is wild-type for flagellar biosynthesis, a flagellum from *S. enterica* strain TMT233a (**b**) and a flagellum from *C. jejuni* (**c**). The hook protein of strain TMT233a consists of the D0, D1, and D2 domains from *S. enterica* FlgE and the D3 and D4 domains inserted from *C. jejuni* FlgE; it therefore builds a flagellar hook that is similar to that of *C. jejuni*. The basal body, the hook, and the filament are indicated by arrows (1), (2) and (3), respectively. (**d**) Model in projection of the hook showing a single ring (11 molecules) of the hook based on the helical parameters of the hook from *S. enterica*. Five molecules in the ring, coloured in grey, are from FlgE of *S. enterica* while the six other molecules, colour-coded from the “N- to C-terminus” in rainbow colours, are from FlgE of *S. enterica* strain TMT233a.

Small volume cultures of strain TMT233a, encoding the FlgE chimera, and the wild-type strain were grown and cell extracts and culture media were harvested. Western blotting, using sera containing anti-FlgE or anti-FliC antibodies was performed and FlgE and FliC proteins were found in the cell pellet and exported into the medium at similar amounts for both strains. Therefore, strain TMT233a appeared to assemble flagella normally (Fig. 3e,f). To further characterize the flagella, flagellar basal bodies (including the hook and filament) were purified following the protocol of Aizawa^16^. Negatively-stained TEM images showed that while the basal body appeared normal (Fig. 4), the hook from strain TMT233a was bulkier than wild type. These data show that an *S. enterica* hook protein that is designed to include *C. jejuni* domains D3 and D4, assembles into a fully-functional hook, with a larger hook diameter. The diameter of the hook with the chimeric FlgE is similar to that of the hook from *C. jejuni*. The average diameter of the hook from strain TMT233a is 25.1 nm compared to 25.9 nm for *C. jejuni* as measured from negatively-stained image taken by electron microscopy (Supplementary Fig. S7). This diameter is also similar to the diameter of the quaternary model of the chimeric hook containing domains D3 and D4 from *C. jejuni* (Fig. 4d). This model was made based on the structure of *S. enterica* polyhook^11^.

A *Salmonella* strain (TMT233b) encoding a chimeric FlgE, FlgE_hyb2_, that has domain D3 only of FlgE from *C. jejuni* inserted into FlgE of *S. enterica* was non-flagellated and non-motile (Fig. 3c). FlgE_hyb2,_ is made of residues Gly233-Ala262 and Ile439-Leu618 of *C. jejuni* FlgE inserted between residues Thr167 and Pro168 of *S. enterica* FlgE. Small volume culture of strain TMT233b was grown, and Western blotting, using serum containing anti-FlgE or anti-FliC antibodies, was performed on both cell extracts and culture media. Although the FlgE chimera, FlgE_hyb2_, was made and exported without degradation, we found no trace of FliC (Fig. 3f). Furthermore, FlgE concentration in the medium was much higher than in wild type cultures. The function of the hook cap protein FlgD is to oversee the polymerization of FlgE proteins at the tip of the growing hook^17^. In the absence of FlgD, FlgE proteins do not assemble to form the hook but are instead secreted in the medium. Although the detailed binding mechanism of FlgD is not known, its N-terminal domain has been shown to bind to the growing hook while its C-terminal domain prevents the secretion of upcoming FlgE proteins in the medium^18^. The reason of the failure in the hook assembly in the strain TMT233b could be due to the disruption in the fixation of FlgD, thus inhibiting the assembly of the hook and resulting in excess secretion of FlgE_hyb2_ protein in the culture medium.

## Discussion

Structures of the flagellar hook proteins described here show that despite the divergence of FlgE proteins throughout the bacterial kingdom and the difference in the length of their primary sequence, FlgE proteins maintain a folding pattern that allows them to form the hook, a flexible universal joint. It is very likely that these hooks have quaternary structures made of 11 protofilaments. The quaternary structure of the hook of *C. crescentus* was shown to consist of 11 protofilaments^3^,^14^. The D3 domains of FlgE from *C. crescentus* and from *C. jejuni* are inserted almost at the same position, but have a totally different 3D structure. The conserved 3D structure of domains D1 and D2 of FlgE from *C. jejuni* and the results from the chimeric strain TMT233a indicate that the hook of *C. jejuni* might have a quaternary structure made of 11 protofilaments. The hook protein, in general, contains conserved segments and folds to make domains D0, D1, and D2. These three domains constitute the basic functional hook and are involved in all the inter-molecular contacts in the hook. Insertions that occur through evolution make additional domains that could bring specificity to the function of the hook. Based on this study, we suggest that the divergence of FlgE protein sequences, which results in hooks of variable diameters and produces different sets of interactions within the hook, must be linked to the swimming environments of bacteria as we have seen in the case *C. jejuni*. A recent study of the flagellar motors of different bacteria has shown that, in the case of *C. jejuni*, the flagellar motor contains extra parts that enable it to produce a much higher torque, compared to the flagellar motor of *S. enterica*^19^. To sustain such high torque and to efficiently transmit rotation to the filament, *C. jejuni* might have developed a stronger or stiffer hook that could have extra connections from domains D3 and D4. Compared to wild-type *S. enterica*, our hybrid strain of *Salmonella*, TMT233a, does not swim as well in normal conditions. The reason could be due to the combination of many factors. One of these factors could be, for example, an instability of the inserted domains in our hybrid strain that could have the effect to increase the drag while swimming. In general, the range of divergence of FlgE proteins and the insertion of large domains in the middle of domain D2, will be fully understood when additional hook structures become available.

## Methods

### Strains and growth conditions

Strains of bacteria and plasmids used in this study are described in Supplementary Table S1. Oligonucleotides used in the strain and plasmid constructions are listed in Supplementary Table S2. *Escherichia coli* and *Salmonella* strains were routinely grown aerobically in Luria-Bertani (LB) medium^20^. For *E. coli*, ampicillin was added to media at 50 μg ml^−1^ as required. For *Salmonella*, ampicillin and tetracycline were added as required, at 100 μg ml^−1^ and 15 μg ml^−1^, respectively.

### Protein expression and purification

The DNA sequence encoding FlgEcc32 was amplified by polymerase chain reaction (PCR) from *C. crescentus* strain CB15 (ATCC 19089) and cloned into a T7 expression vector modified from pET-28b(+) (Novagen). Primers used for PCR are listed in Supplementary Table S1. Standard procedures for DNA manipulation were followed^20^. The construct was then transformed into BL21(DE3) cells (Novagen) for expression. SeMet-substituted FlgEcc32 was expressed in methionine-auxotrophic *E. coli* strain B834(DE3)/pRARE cells (Novagen) using SeMet core medium (Wako Pure Chemical Industries). BL21(DE3) cells were grown in 5 L Luria Bertani (LB) medium containing 40 μg mL^−1^ kanamycin at 310 K in a 10 L fermenter. Protein expression by the cells was induced with 0.5 m*M* isopropyl β–D-1-thiogalactopyranoside (IPTG) at an OD_600_ of 0.5 and cultivation continued for 3 h at 310 K. The cultured cells were harvested by centrifugation at 8000 × *g* for 15 min. The cell pellet was suspended in 50 mL lysis buffer [20 m*M* Tris-HCl pH 8.0, 10 m*M* NaCl] and disrupted by sonication at 277 K. The crude lysate was centrifuged at 100,000 × *g* for 1 h at 277 K. Supernatant was loaded onto a HiTrap Q FF column (GE Healthcare) pre-equilibrated with 10 m*M* NaCl, 20 m*M* Tris-HCl at pH 8.0. Protein was eluted from the column with elution buffer (20 m*M* Tris-HCl pH 8.0, 500 m*M* NaCl) using a linear gradient (10–500 m*M*) of NaCl. Fractions were pooled and concentrated for gel-filtration chromatography. After concentration, the protein was applied onto a Superdex 75 prep-grade (16/60) gel filtration column (GE Healthcare) equilibrated with 10 m*M* Tris-HCl pH 8.0. Purified FlgEcc32 was concentrated to 35 mg ml^−1^ using a Centriprep centrifugal filter device (Millipore).

### Crystallization, data collection and structure determination

Initial crystallization screening was performed at 293, 288, 283 and 278 K by the sitting-drop vapor-diffusion technique using an automated nanoliter liquid-handling system (Mosquito, TTP LabTech) in a 96-well plate. The drops consisted of 150 nL protein solution (12 mg mL^−1^) and 150 nL reservoir solution and were equilibrated against 100 μL reservoir solution using commercial screening kits from Hampton Research and Emerald BioSystem. After 6 days, FlgEcc32 crystals were observed in reservoir conditions containing of 0.1 *M* CHES pH 9.5, 1.0 *M* Sodium citrate at 288 K and 293 K respectively. The optimal crystallization buffer was found to be 0.1 *M* TAPS pH 9.0, 1 *M* Potassium citrate, 5% (v/v) 1,5-pentanediol and a protein concentration of 5 mg mL^−1^.

Crystallization of the FlgEcc32 protein was performed using the hanging-drop vapour-diffusion method (with a drop consisting of 2 μL protein solution and 2 μL reservoir solution equilibrated against 1 mL reservoir solution) at 293 K.

Crystals were soaked in a cryo-protectant solution that consisted of the reservoir solution with 20% (v/v) glycerol. Crystals were mounted on a cryo-loop and flash-cooled in liquid nitrogen.

X-ray diffraction data were collected using synchrotron radiation (wavelength 0.9 Å) and MAR225HE CCD detector on beamline BL44XU at the 8-GeV Super Photon ring (SPring-8) in Harima, Japan. All images were collected at 100 K using a 1° oscillation angle per frame. These crystals diffracted to about 1.8 Å resolution and belonged to space group *P*2_1_ with unit cell parameters a = 54.5 Å, b = 61.3 Å, c = 102.8 Å, β = 91.4° with 2 molecules in the asymmetric unit.

SeMet-containing crystals of were obtained under the same conditions. Data collection statistics are summarized in Table 1.

Preparation, crystallization, and X-ray data collection of the FlgEcj79 fragment of *C. jejuni* NCTC 11168 FlgE have been previously detailed^21^. These crystals diffracted to about 2.45 Å resolution and belonged to space group *P*2_1_ with unit cell parameters a = 75.3 Å, b = 173.2 Å, c = 146.9 Å, β = 102.7° with four molecules in the asymmetric unit.

X-ray diffraction data from FlgEcc32 and from FlgEcj79 data were reduced by *hkl2000*^22^. Phases were obtained from sets of SAD and MAD, respectively, and calculated with *Autosol*^23^. Partial tracing of the main chain was done with *Autobuild*^24^ and completed with *Coot*^25^. FlgEcc32 and FlgEcj79 were refined to resolutions of 1.84 Å and 2.45 Å, respectively, with *phenix.refine*^26^.

### Construction of *Salmonella flgE* mutant strains

SJW1103 is an *S. enterica* strain that is wild type for chemotaxis and motility^27^. Two FlgE chimera mutant strains were derived from SJW1103 that either encoded a *flgE* gene with codons for the *C. jejuni* FlgE D3 and D4 domains inserted (strain TMT233a) or that encoded a *flgE* gene with codons for the *C. jejuni* FlgE D3 domain inserted (strain TMT233b). The bacteriophage Lambda-Red homologous recombination method of Karlinsey was used to make the strains^28^. Briefly, the first strain made was TMTflgEtetRA, which bore an inserted tetracycline-resistance cassette in the *flgE* gene. The tetracycline-resistance cassette was generated by PCR using genomic DNA from strain TT13206 as template and primers Fd-flgE-tetRA and Rv-flgE-tetRA. The tetracycline-resistance cassette was integrated onto the chromosome using Lambda Red homologous recombination and recombinant mutant strains were selected on media containing tetracycline.

Strain TMT233a was made from TMTflgEtetRA bearing codons encoding the D3 and D4 domains from *C. jejuni* FlgE inserted into the *flgE* gene. Codons 233 to 618 of the *C. jejuni* NCTC 11168 *flgE* gene were amplified by PCR using genomic DNA as template and primers Fd-CampyflgE233 and Rv-CampyflgE619. Strain TMT233b was also made from TMTflgEtetRA, bearing codons encoding the *C. jejuni* FlgE D3 domain only inserted into the *flgE* gene. The D3 domain is encoded by codons 233 to 262 and codons 439 to 618 of the *C. jejuni* NCTC 11168 *flgE* gene. A PCR fragment encoding codons 233 to 262 fused to codons 439 to 618, for insertion into the *Salmonella flgE* gene by homologous recombination, was made using overlap extension PCR^29^. Codons 233 to 262 of the *C. jejuni* NCTC 11168 *flgE* gene were amplified by PCR using genomic DNA as template and primers Fd-CampyflgE233 and Rv-CampyflgE262. Codons 439 to 618 of the *C. jejuni* NCTC 11168 *flgE* gene were amplified by PCR using genomic DNA as template and primers Fd-CampyflgE439 and Rv-CampyflgE619. The PCR product of codons 233 to 262 and the PCR product of codons 439 to 619 were used as template with primers Fd-CampyflgE233 and Rv-CampyflgE619 in an overlap extension PCR reaction. Codons for *C. jejuni* FlgE D3 and D4 domains, and only the D3 domain, were integrated onto the chromosome of strain TMTflgEtetRA using Lambda RED homologous recombination and recombinant mutant strains were selected on media containing 12 μg mL^−1^ fusaric acid as decribed by Karlinsey^28^. Standard procedures for DNA manipulation were followed^20^.

### Motility assays

For *S. enterica*, soft-tryptone agar plates containing tryptone (10 g L^−1^), sodium chloride (7 g L^−1^), and agar (3.5 g L^−1^) were stab inoculated with freshly-grown colonies and incubated at 30 °C as previously described^30^. Experiments were repeated at least six times for each strain.

### Flagellar protein export assay

Assays of *Salmonella* FlgE and FliC protein export were performed essentially as described previously^31^. Briefly, cultures of *Salmonella* grown at 37 °C in LB medium were centrifuged to obtain cell pellets and culture supernatants. Cell pellets were normalized to a constant cell density and suspended in sodium dodecyl sulfate (SDS)-loading buffer. Proteins secreted into the medium were precipitated with 10% (v/v) trichloroacetic acid, suspended in Tris/SDS loading buffer, and heated at 95 °C for 15 min. Then they were separated by electrophoresis in 12.5% SDS-polyacrylamide gel electrophoresis (PAGE) gels and transferred to polyvinylidene fluoride (PVDF) membranes. Western-blotting was performed using a WesternBreeze chromogenic immunodetection kit (Thermo Fisher Scientific, USA) for detection of rabbit primary antibodies. Serum containing anti-FlgE polyclonal antibodies was diluted 1:10,000 and serum containing anti-FliC antibodies was diluted 1:20,000.

### Examination of whole cells and purified flagellar hook-basal bodies by electron microscopy

*Salmonella enterica* cells were examined by transmission electron microscopy similarly as previously described^32^. Cells were immobilized on Mextaform HF-34 200-mesh carbon-coated copper grids, and stained directly with 1% (w/v) uranyl acetate (pH 4). Grids were examined using a Jeol JEM-1230R transmission electron microscope (JEOL, Tokyo, Japan) at 100 kV and 2,000 to 5,000x magnification. At least 50 cells were examined for each strain. Hook-basal body particles were isolated as described by Aizawa^16^ with slight modifications. Prepared samples were negatively stained with 1% (w/v) uranyl acetate (pH 4) and micrographs were recorded with a JEM-1230R transmission electron microscope at 100 kV and 20,000 to 30,000x magnification.

### Measurements of free-swimming speeds of motile *Salmonella* cells

*Salmonella* strains were grown in LB medium to an OD_600_ nm 1.0 at 37 °C. Cells were harvested by low-speed centrifugation (1000 × g for eight minutes at room temperature) and suspended in an equal volume of motility buffer (10 mM potassium phosphate pH 7.0; 0.1 mM EDTA; 10 mM DL-sodium lactate), as described previously^33^. Motility buffer was supplemented with Ficoll PM400 (GE Healthcare, USA) to increase viscosity, as required^34^. Cells were examined immediately using dark-field optics at 100x magnification (1000x with the camera) at room temperature, approximately 23 °C. Several 30 second videos were recorded using Olympus cellSens Microscope Imaging Software (Olympus Corporation, Japan). Compressed avi files were saved as decompressed avi files with ICY software (http://icy.bioimageanalysis.org/) and imported into Fiji software (https://fiji.sc/). Trajectories and velocities (pixels/frame) of >40 bacteria were calculated with the “TrackMate” plugin (http://imagej.net/TrackMate). Velocities were converted to μm/sec values. Immobile cells or very slow cell tracks (swimming velocity <3 μm/sec) were excluded from the data analysis. Tracks with fewer than 10 spot detections were also not included. Experiments were repeated independently at least twice, and a representative data set is shown.

## Additional Information

**Accession codes:** Atomic coordinates have been deposited in the Protein Data Bank under accession code 5AY6 for FlgE from *C. crescentus* and under accession code 5AZ4 for FlgE from *C. jejuni.* The X-ray diffraction data were collected in Spring-8 (Harima, Japan) under the proposals number 2009B2106, 2010A6534, 2011A1466, 2011B2014, 2013A1880 and 2013A6845. (Interactive 3D views of the structures reported here: http://proteopedia.org/w/Samatey/3).

**How to cite this article**: Yoon, Y.-H. *et al*. Structural insights into bacterial flagellar hooks similarities and specificities. *Sci. Rep.* **6**, 35552; doi: 10.1038/srep35552 (2016).

## Supplementary Material

Supplementary Information

Supplementary Movie S1

## Figures and Tables

**Table 1:** Data collection and refinement statistics for FlgEcc32 and FlgEcj79 crystals.

	FlgEcc32		FlgEcj79		
	Native	SeMet peak	Native	SeMet peak	SeMet edge
Data collection					
Space group	*P*2_1_	*P*2_1_	*P*2_1_	*P*2_1_	*P*2_1_
Cell dimensions (Å,°)					
*a*	54.54	54.84	75.46	75.45	75.45
*b*	61.30	61.50	173.54	173.76	173.76
*c*	102.86	103.11	147.09	147.69	147.69
*β*	91.37	91.70	102.66	102.98	102.98
Wavelength (Å)	0.9	0.9791	1.0	0.9791	0.9794
Resolution (Å)	50.0–1.84 (1.87–1.84)	50.0–2.00 (2.03–2.00)	41.0–2.45 (2.49–2.45)	37.69–2.60 (2.74–2.60)	37.71–2.60 (2.74–2.60)
*R*_merge_	9.4 (30.4)	8.8 (20.1)	12.6 (91.4)	13.0 (45.6)	13.7 (52.1)
Mean <*I/σ (I)*>	13.6 (2.2)	32.3 (10.5)	24.2 (2.7)	8.0 (3.0)	7.4 (2.7)
Completeness (%)	97.6 (92.6)	100 (99.3)	99.5 (99.0)	99.9 (99.9)	100 (100)
Redundancy	3.0 (2.3)	5.1 (5.0)	11.2 (10.9)	3.8 (3.7)	3.8 (3.8)
Refinement					
Resolution (Å)	19.8–1.84		25.0–2.45		
No. reflections	59519		133379		
*R*_work_/*R*_free_	17.6/22.2		20.7/25.4		
No. atoms					
Protein	4402		21712		
Water	1114		828		
*B*-factors					
Protein	14.4		60.7		
Water	25.1		43.1		
R.m.s deviations					
Bond length (Å)	0.005		0.005		
Bond angle (°)	0.93		0.91		
