# Supplementary material for: Structural insights into bacterial flagellar hooks similarities and specificities
Source: Sci Rep. 2016 Oct 19;6:35552. doi: 10.1038/srep35552 (PMC5069557; doi:10.1038/srep35552)
Supplement: Supplementary Information [file srep35552-s1.pdf]

Supplementary information

Structural insights into bacterial flagellar hooks similarities and specificities

Young-Ho Yoon, Clive S. Barker, Paula V. Bulieris, Hideyuki Matsunami, Fadel A. Samatey*

Affiliation:

Trans-Membrane Trafficking Unit, Okinawa Institute of Science and Technology Graduate University, 1919-1, Tancha, Onna, Kunigami, Okinawa 904-0495, Japan.

* Address correspondence to Fadel A. Samatey: bakary.macy.samatey@gmail.com

Table S1. Strains of bacteria and plasmids.

Strain or plasmid	Genotype or description	Reference or source
Escherichia coli		
NovaBlue	K-12 strain derivative for routine molecular cloning applications	Novagen, USA
NEB 5-alpha	K-12 strain derivative used in conjunction with Gibson assembly cloning kits	New England Biolabs, USA
BL21(DE3)	*E. coli* B T7 protein-expression strain; Lon and OmpT protease deficient	Novagen, USA
B834(DE3)/pRARE	*E. coli* B T7 protein-expression strain; OmpT protease deficient; methionine auxotroph; Rare codon expression; Cam^R	Novagen, USA
***Salmonella enterica* serovar Typhimurium**		
SJW1103	Wild-type for motility and chemotaxis; LT2 (*fljB*-off) Δ(*hin-fljAB*)	(27)
JR501	For converting plasmids to *Salmonella* compatibility. Restriction minus, modification plus	(35)
TT13206	LT7 *phoN51*::Tn*10-11*(Tet^R)	(36)
TMTflgEtetRA	*flgE*(*1-168*)*22591*::*tetRA*::*flgE*(*169-404*)	This study
TMT233a	*flgE*(*1-168*)*22592*::*flgE*$_{C.\ jejuni\ \mathrm{NCTC\ 11168}}$(*233-618*)::*flgE*(*169-404*)	This study
TMT233b	*flgE*(*1-168*)*22593*::*flgE*$_{C.\ jejuni\ \mathrm{NCTC\ 11168}}$(*233-262*:: *439-618*)::*flgE*(*169-404*)	This study
**Plasmid**		
pET-28b(+) + FlgEcc32	pET-28b(+) derivative carrying codons of the *Caulobacter crescentus flgE* gene to express FlgEcc32	This study
pKD46	Bacteriophage λ Red expression plasmid, Amp^R	(37)

Table S2. Oligonucleotide primers.

Name	Sequence (5' to 3')	Template
Primers used to make FlgEcc32 expression vector by Xho I and BamH I restriction enzyme digestion of DNA followed by ligation using T4 DNA ligase:		
Fd-flgEcc32	gggcccctcgagatg**GCCGAGAAGACCACCCGC**	*C. crescentus* CB15 genomic DNA
Rv-flgEcc32	gggcccggatcctta**GCCGTTGGTGTTCACCGAC**	*C. crescentus* CB15 genomic DNA
Primers used to make strain TMTflgEtetRA:		
Fd-flgE-tetRA	gatcaacctgaactcaacggaccctgtaccgtctaaaacg**TTAAGACCCACTTTCACATT**	TT13206 genomic DNA
Rv-flgE-tetRA	ctttttgttatacgaatccgcatcactcacgctaaaggg**CTAAGCACTTGTCTCCTG**	TT13206 genomic DNA
Primers used to make strain TMT233a and strain TMT233b:		
Fd-CampyflgE233	gatcaacctgaactcaacggaccctgtaccgtctaaaacg**GGTGTGGATGCGGGATCACTT**	*C. jejuni* NCTC 11168 genomic DNA
Rv-CampyflgE619	ctttttgttatacgaatccgcatcactcacgctaaaggg**AAGTTGTTCGCTTTCTTTGATCTGATTTC**	*C. jejuni* NCTC 11168 genomic DNA
Primers used to make strain TMT233b:		
Fd-CampyflgE439	atcttatgcagatgcaa**TAATAACAGCACATAAATATATTTATAGTTCAAAC**	*C. jejuni* NCTC 11168 genomic DNA
Rv-CampyflgE262	tatgtgctgttatta**TTGCATCTGCATAAGATACCCAAATTC**	*C. jejuni* NCTC 11168 genomic DNA

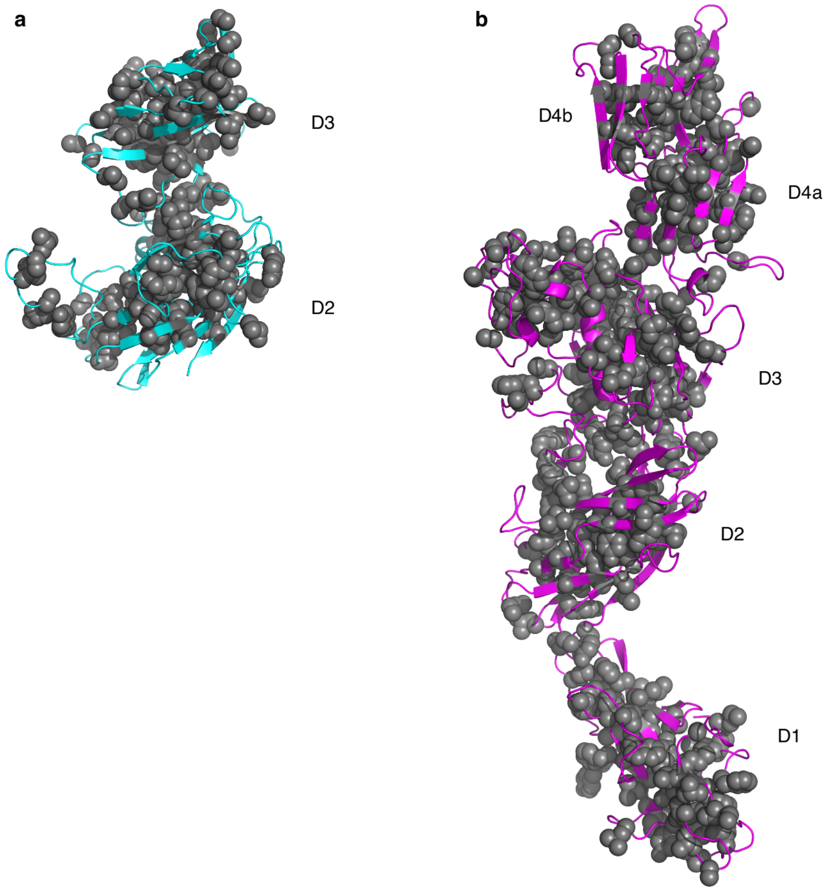

Supplementary Figure S1. Domain delimitation in *C. crescentus* and *C. jejuni*.

Hydrophobic amino acids, represented by gray spheres, are superimposed on a cartoon representation of FlgEcc32 (cyan) (a) and of FlgEcj79 (purple) (b). Four distinct domains are identifiable: D1, D2, D3, D4. D4 can be divided into two sub-domains, D4a and D4b.

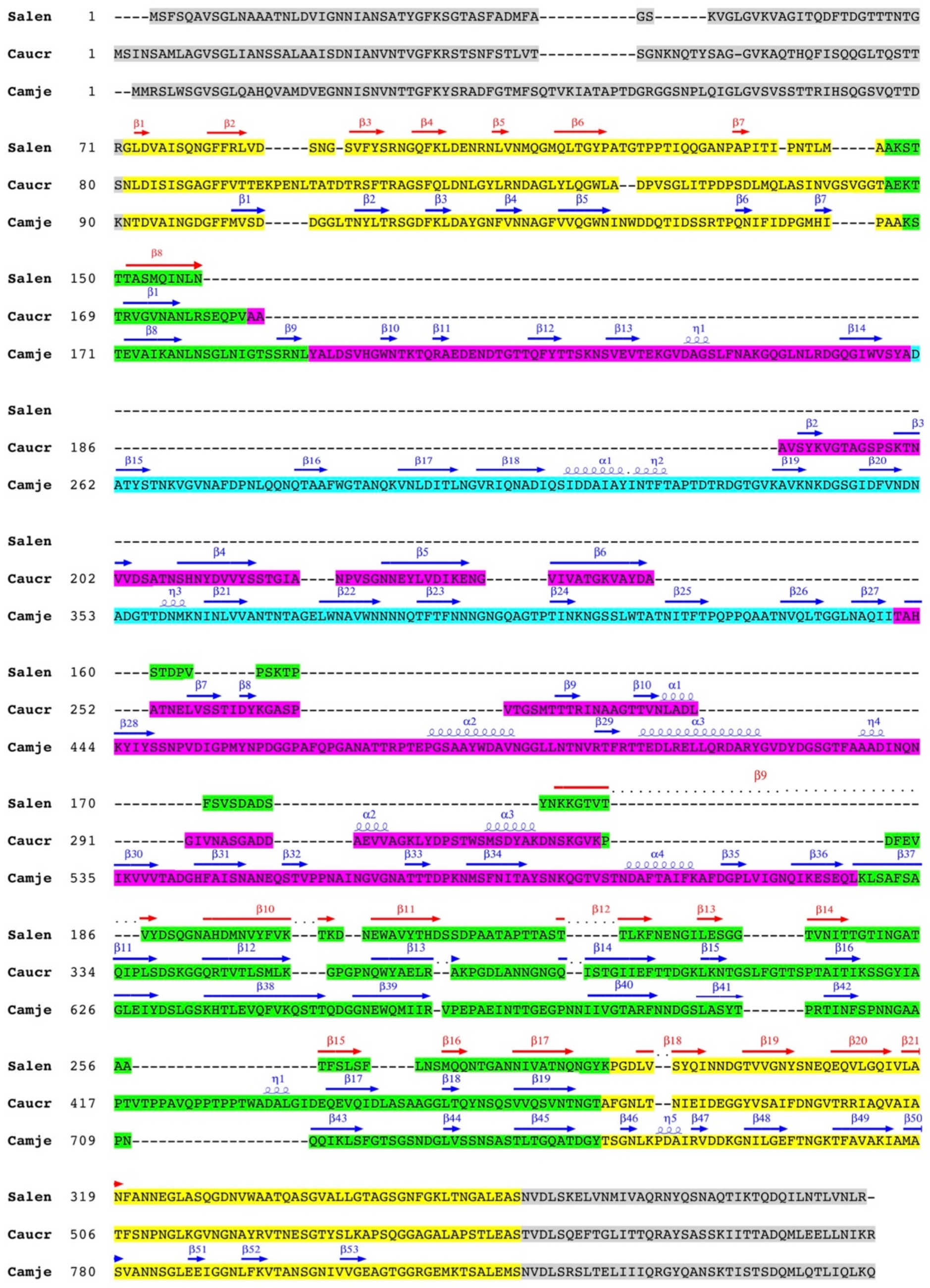

Supplementary Figure S2. Sequence alignment of full-length FlgE proteins of *S. enterica, C. crescentus* and *C. jejuni*. Salen = *S. enterica* serovar Typhimurium LT2 FlgE (NCBI Reference Sequence: NP_460148.1); Camje = *C. jejuni* subsp. *jejuni* NCTC 11168 FlgE (NCBI Reference Sequence: YP_002345095.1); and Caucr = *C. crescentus* CB15 FlgE (NCBI Reference Sequence: NP_419718.1). Domain D0, which participates in the coiled-coil interaction, is in gray. Domains D1 and D2, found in each of these bacteria, are in yellow and green, respectively. Domain D3, found in *C. jejuni* and *C. crescentus*, is in red. Domain D4, which exists only in *C. jejuni*, is in cyan. Amino acid sequences of proteins were aligned using Clustal Omega[38], and secondary structure rendering used ESPript 3.0[39].

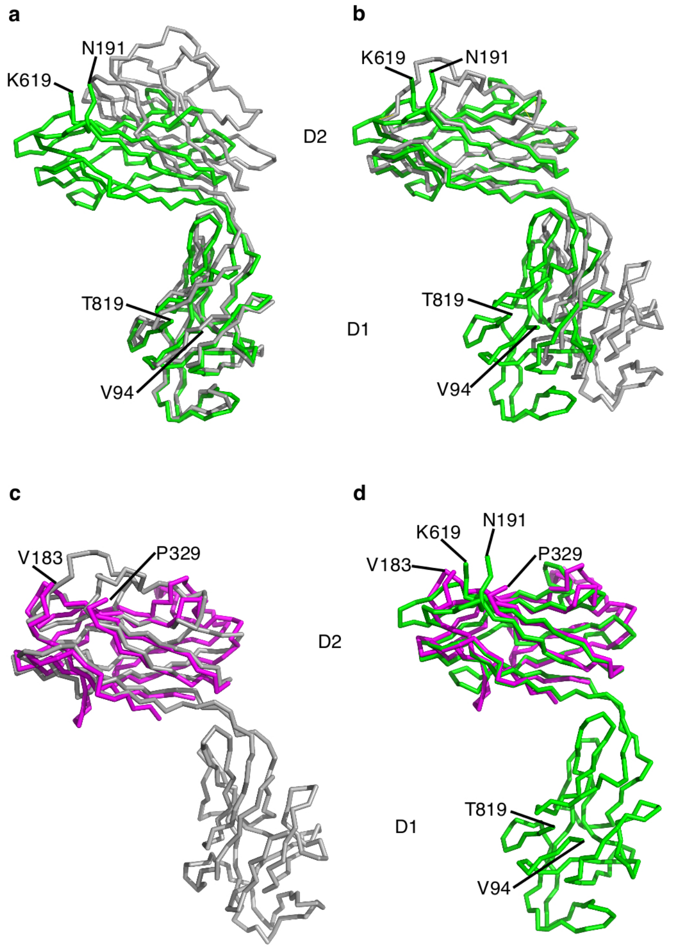

Supplementary Figure S3. Structural comparison of FlgE from *C. crescentus*, *C. jejuni,* and *S. enterica*. Alignment of domains D1 (RMSD 1.4 Å) (a) and domains D2 (RMSD 1.6 Å) (b) of FlgE from *C. jejuni* (green) and *S. enterica* (gray). N191 and K619 indicate where the D3 and D4 domains were removed from the *C. jejuni* chain. Alignment of domains D2 of FlgE from *C. crescentus* (magenta) and *S. enterica* (gray) with an RMSD of 1.7 Å (c) and from *C. crescentus* (magenta) and *C. jejuni* (green) with an RMSD of 0.9 Å (d). V183 and P329 show where the D3 domain was removed from the *C. crescentus* chain.

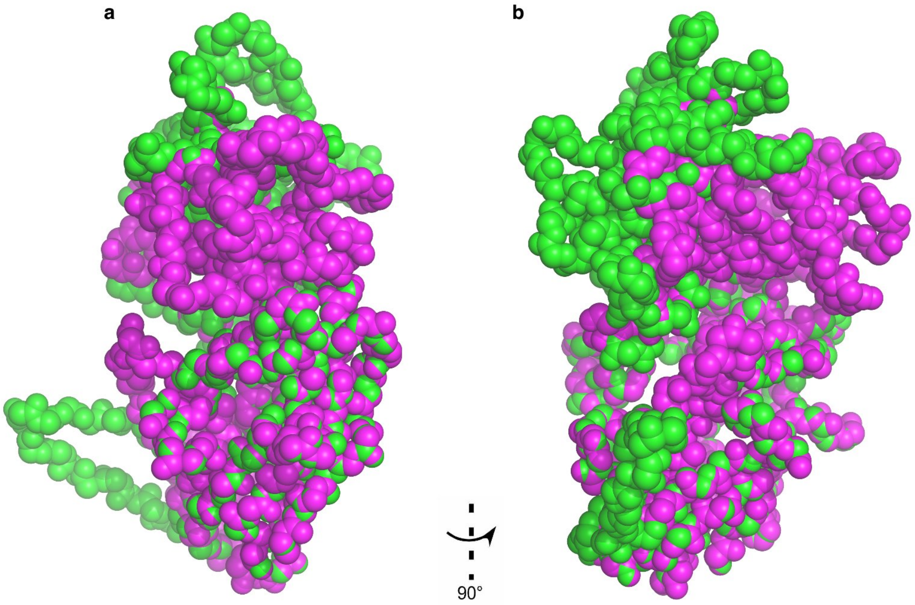

Supplementary Figure S4. Structure alignment.

Superimposition of structures of FlgE protein of *C. crescentus* in the crystal (in green) and in the hook after docking (in magenta). The alignment is based on domain D2.

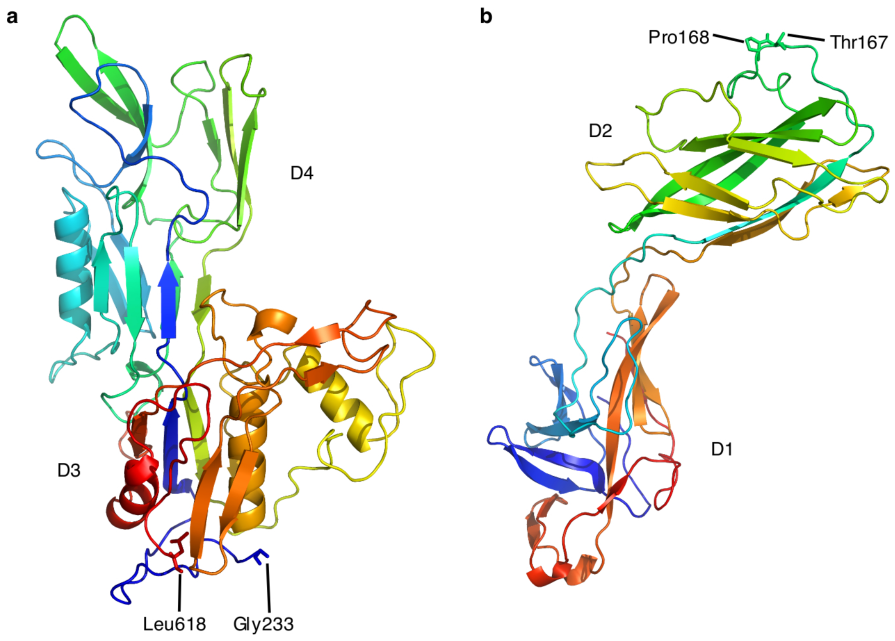

Supplementary Figure S5. Construction of FlgEhyb1.

The chimeric FlgE protein, FlgEhyb1, was designed by inserting domains D3 and D4 from FlgE of *C. jejuni* (a) between Thr167 and Pro168 located in domain D2 of FlgE of *S. enterica* (b). Domains D1 and D2 are common to all hook proteins. The hook protein of *C. jejuni*, with additional domains D3 and D4, is larger than a normal hook protein composed only of domains D1 and D2. It is surprising that domains D3 and D4 can be inserted with no disturbance to the remainder of the quaternary structure of the hook and without altering its function.

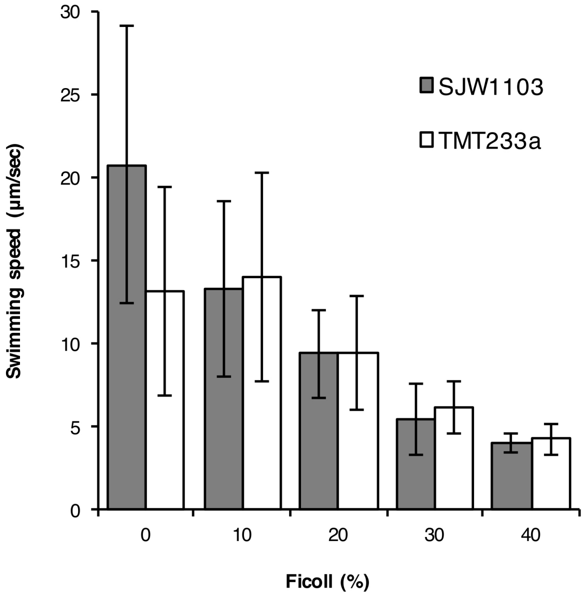

Supplementary Figure S6. Free-swimming speed of the wild-type *S. enterica* strain SJW1103 and the FlgE chimera mutant strain TMT233a in solutions of increasing viscosity.

Solution viscosity was increased using increasing concentrations of ficoll PM400. Swimming speed was determined for more than 40 cells using a microscope fitted with dark-field optics and a video recording system. Mean swimming speeds and standard deviations are shown. Measurements were done at around 23˚C.

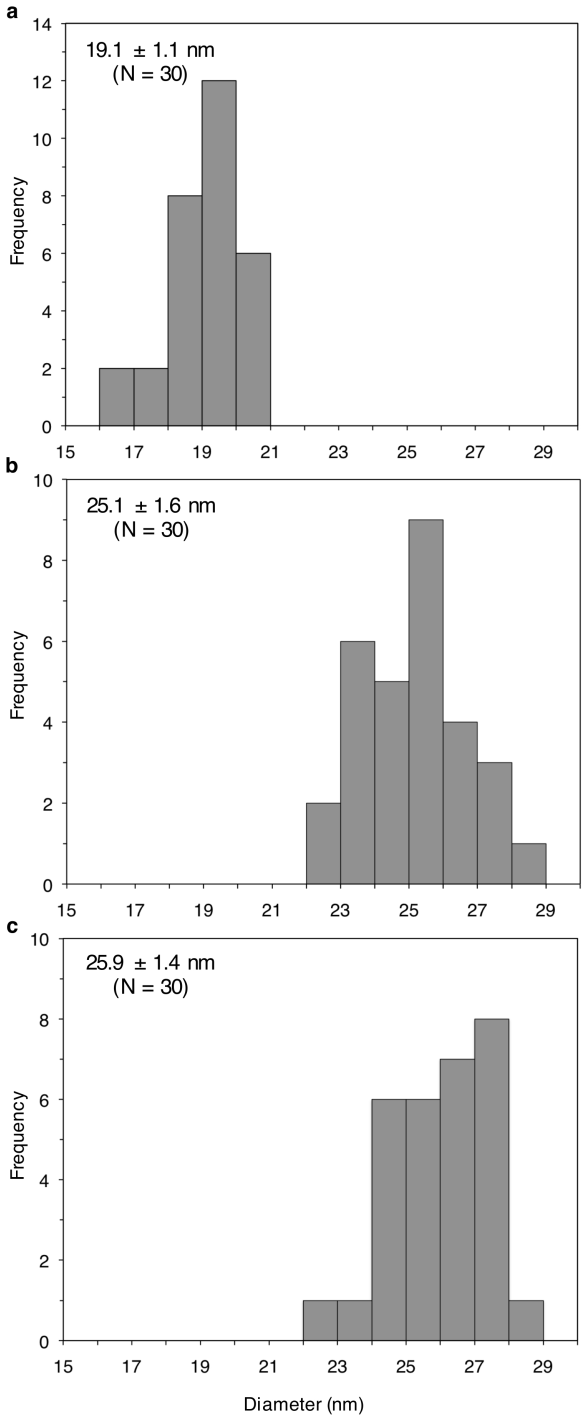

Supplementary Figure S7. Diameter distribution of the hooks.

S. enterica strain wild-type SJW1103 (a), TMT233a (b), and *C. jejuni* 81116 (c). The hook diameters were measured from negatively-stained images obtained by electron microscopy using software ImageJ[40]. Average hook diameter sizes and the standard deviation values are shown in each panel.

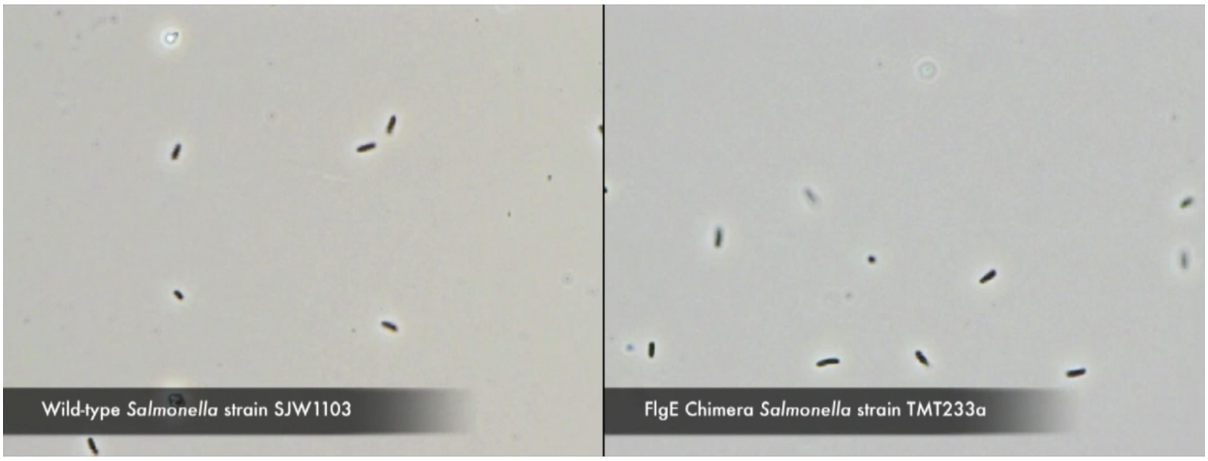

Supplementary Movie S1. Motility of *S. enterica* wild-type and strain TMT233a.

The left panel displays the motility of *S. enterica* strain SJW1103 (wild type). The right panel shows strain TMT233a, which has a hybrid hook made of *S. enterica* FlgE grafted with domains D3 and D4 of *C. jejuni* FlgE.
